# The Transmembrane Protein of the Human Endogenous Retrovirus - K (HERV-K) Modulates Cytokine Release and Gene Expression

**DOI:** 10.1371/journal.pone.0070399

**Published:** 2013-08-07

**Authors:** Vladimir A. Morozov, Viet Loan Dao Thi, Joachim Denner

**Affiliations:** Robert Koch Institute, Berlin, Germany; Plymouth University, United Kingdom

## Abstract

Numerous copies of endogenous retroviruses are present in the genome of mammals including man. Although most of them are defective, some, e.g., the human endogenous retroviruses HERV-K, were found to be expressed under certain physiological conditions. For instance, HERV-K is expressed in germ cell tumours and melanomas as well as in the placenta. Most exogenous retroviruses including the human immunodeficiency virus HIV-1 induce severe immunodeficiencies and there is increasing evidence that the transmembrane envelope (TM) proteins of these retroviruses may be involved. We show here that HERV-K particles released from a human teratocarcinoma cell line, a recombinant TM protein and a peptide corresponding to a highly conserved so-called immunosuppressive domain in the TM protein of HERV-K inhibit the proliferation of human immune cells, induce modulation of the expression of numerous cytokines, and modulate the expression of cellular genes as detected by a microarray analysis. The changes in cytokine release and gene expression induced by the TM protein of HERV-K are similar to those found previously induced by the TM protein of HIV-1. These data suggest that the mechanism of immunosuppression may be similar for different retroviruses and that the expression of the TM protein in tumours and in the placenta may suppress immune responses and thus prevent rejection of the tumour and the embryo.

## Introduction

In the last years the expression of human endogenous retroviruses (HERV) in tumours and physiologically healthy tissues was intensively studied [1]–[3]. HERV-K is one of the few human endogenous retroviruses with intact open reading frames for all viral proteins [4], [5]. Non-infectious virus-like particles and viral proteins have been found in human germ line tumours [6]–[8] and melanomas [9]–[11]. In addition, we have recently shown that HERV-K proteins are expressed in the human placenta [12]. Antibodies against HERV-K and its TM protein were found in some tumour patients and pregnant women [1], [10], [11]. Genes of other human endogenous retroviruses such as syncytin 1 (the envelope protein of HERV-W) [13]–[15] and syncytin 2 (the envelope protein of HERV-FRD) [16], [17] were also found to be expressed in the placenta. Syncytins play a key role in generating the syncytiotrophoblast cell layer during placentogenesis by inducing cell-cell fusion. In animals, the same function is fulfilled by syncytin-A and syncytin-B in the placenta of mice [18], [19], by syncytin-Ory1 in rabbits [20], syncytin-like env-Cav1 in guinea pigs [21], syncytin-Car1 in cats and dogs [22], and by enJSRV in sheeps [23]. It is important to note that these endogenous retroviruses are not related and that each mammalian species utilises (“enslaved”) a different endogenous retrovirus.

Retrovirus infections are frequently associated with tumours and/or immunodeficiencies. There is increasing evidence that the transmembrane envelope (TM) proteins of retroviruses including HIV-1 may contribute to the retroviral immunosuppression [3], [24]–[26]. Expression of retroviral TM proteins on tumour cells (originally only growing to tumours in immunocompromised animals), allowed these cells to grow in immunocompetent animals, indicating their immunosuppressive property *in vivo* [24]. Recently we showed that single mutations in the so called immunosuppressive (isu) domain of the TM protein gp41 of HIV-1 per se or in the context of replication competent virus particles abrogated the modulation of the cytokine release and gene expression in human peripheral blood mononuclear cells (PBMCs) induced by gp41 [27]. Furthermore, when studying over 2000 HIV-1 sequences from infected individuals, no sequences with abrogating mutations were found, indicating that mutated, non-immunosuppressive HIV-1 may be eliminated by the immune system [27]. In addition, a peptide corresponding to the isu domain of gp41 inhibited human PBMCs and modulated the expression of certain cytokines and other genes as shown in a comparative transcriptome analysis [28].

Here we demonstrate that the recombinant TM protein of HERV-K produced in yeast cells and a synthetic peptide corresponding to a conserved domain of the TM protein inhibit the proliferation of human and mouse immune cells, modulate the release of different cytokines such as the immunosuppressive IL-10 and changes the expression of numerous genes in human PBMCs. In addition, HERV-K particles produced by human teratocarcinoma cells also induced IL-10 release. Since the TM protein of HERV-K was found to be expressed in germ line tumours and melanomas as well as in the placenta, its expression may contribute to the growth of the tumour and the protection of the embryo.

## Results

### The TM Protein of HERV-K Contains an Immunosuppressive (isu) Domain

**Figure 1:**
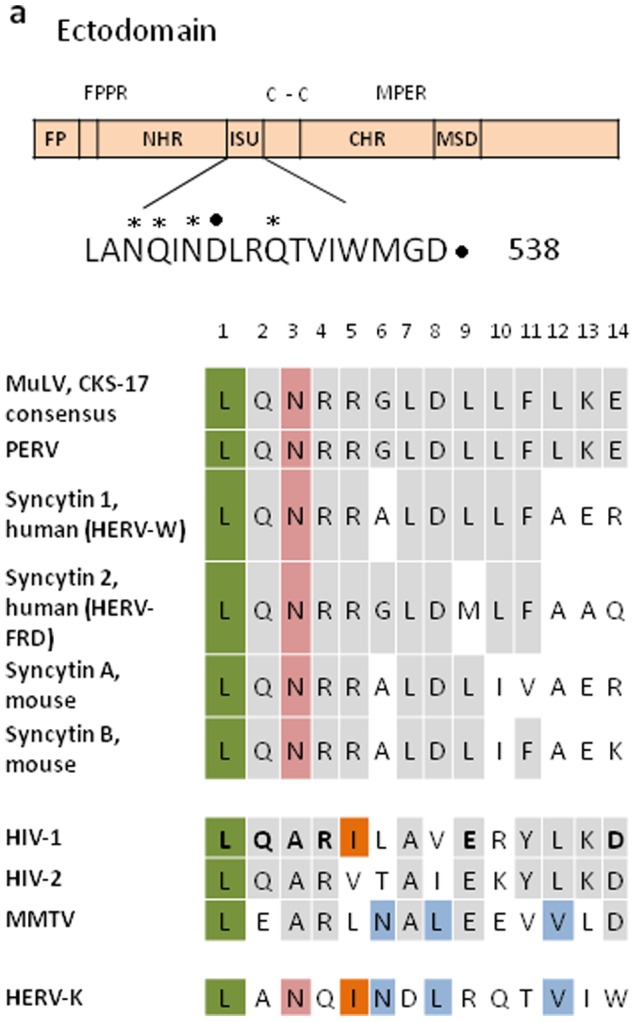
Localisation and sequence of the immunosuppressive (isu) domain of the TM protein of HERV-K (accession number Q69384). **a,** Functional domains of the TM protein: FP, fusion peptide; FPPR, fusion peptide proximal region; NHR, N-terminal helical region; ISU, isu domain; C-C, cystein-cystein loop; CHR, C-terminal helical region; MPER, membrane proximal external region; MSD, membrane spanning domain. In the amino acid sequence of the isu domain stars (*) indicate NH_2_ groups, points (.) mark COOH groups relevant for polymerisation. **b,** Sequence comparison of the core (1 corresponds to the amino acid 552, 14 to 535, acc. Nr. Q69384) of the immunosuppressive domain of different retroviruses (MuLV, murine leukaemia virus; CKS-17 consensus, consensus sequence of the gammaretroviruses PERV, porcine endogenous retrovirus; HERV-K, -W, -FRD; human endogenous retroviruses-K, -W, -FRD; HIV-1, -2, human immunodeficiency viruses - 1, -2¸ MMTV, mouse mammary tumour virus; HERV-K, human endogenous retrovirus-K). Amino acids identical to that in the first sequence of each group are indicated gray. In addition, amino acids present in all retroviruses are marked green, in all gammaretroviruses and HERV-K pink, in HIV-1 and HERV-K orange and in MMTV and HERV-K blue. In the sequence of HIV-1 the amino acids with high importance are shown in bold, mutation of these amino acids totally abrogated the activity to induce IL-10 [27].

The structure of the TM protein of HERV-K is similar to that of other retroviruses (Figure 1a). The so-called immunosuppressive (isu) domain, which is conserved among retroviruses (Figure 1b), is located in proximity to the N-terminal end of the Cys-Cys loop. Retroviruses cluster in different groups according to the sequence of the isu domain. One group includes the gammaretroviruses, another the lentiviruses with the human (HIV-1, HIV-2) and simian (SIV) immunodeficiency viruses. HERV-K is different compared with these groups and contains amino acids from both groups as well as from another betaretrovirus, the mouse mammary tumour virus MMTV (Figure 1b). More than 80% of the published HERV-K sequences contain the isu domain LANQINDLRQTVIW. In rare cases the sequences LASQINDLRQTVIW and LANQINDLRQSVTW were found (Table S1), indicating a high conservation of this domain in all HERV-K proviruses in the human genome.

### Characterisation of the TM Protein of HERV-K Produced in Yeast

To avoid contaminations with bacterial endotoxin, which may modulate the cytokine release when added to human PBMCs, the ectodomain of the TM protein of HERV-K was expressed in *H. polymorpha* and purified by affinity chromatography. Three molecules were expressed in transformed cells: 32 kDa, 30 kDa, and 20 kDa (Figure S1a). All three reacted with an antiserum induced by immunisation with the recombinant TM protein of HERV-K produced in *E. coli* and a serum against the His tag (Figure S1b, c). Since the predicted molecular weight of the protein backbone is nearly 17 kDa, we conclude that the proteins are glycosylated to a different extend. Noteworthy, most probably due to the glycan chains, the TM protein produced in yeast reacted less with the antiserum than the TM protein produced in *E. coli* that was used for immunisation (Figure S1). As a matter of fact, at least five epitopes were recognised in the TM protein [11], and one epitope contained one of the four predicted glycosylation sites (Figure S1d).

### The TM Protein of HERV-K and the Corresponding isu Peptide Inhibit Activation of PBMCs

**Figure 2:**
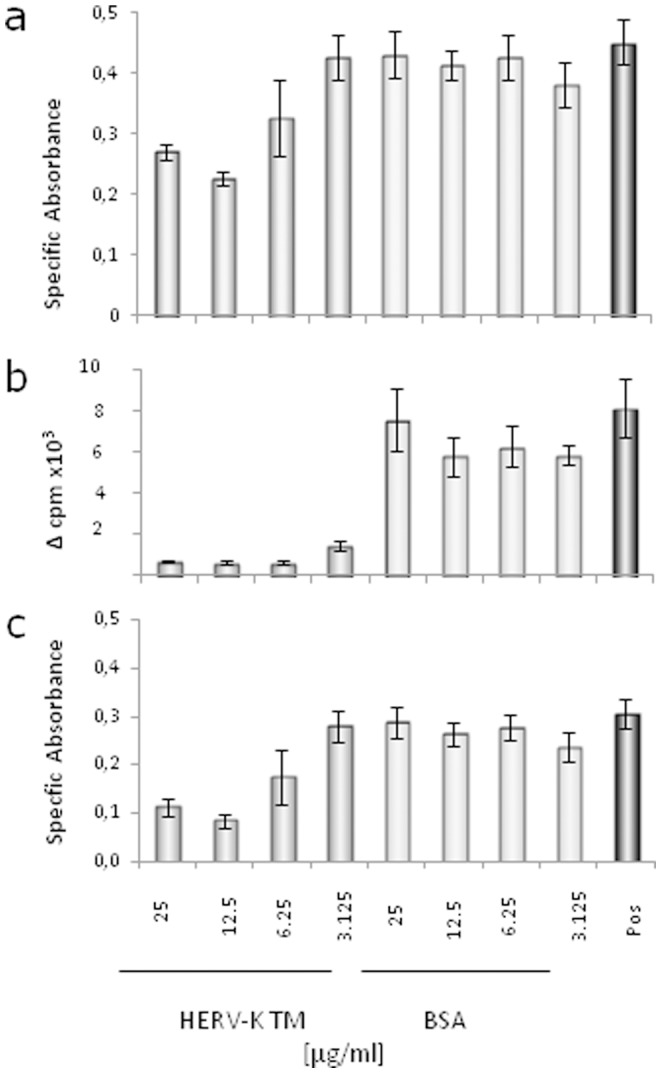
Influence of the recombinant TM protein of HERV-K on the proliferation of ConA-stimulated PBMCs from one healthy human blood donor (a, b) or murine splenocytes (c). Cell proliferation was measured using the Alamar blue assay (**a, c**) or by ^3^H-thymidine incorporation (**b**) (mean±SD; n = 3). ^3^H-thymidine was added on day three and cells were then harvested one the next day and the counts per minute were determined, dark gray – isu peptide-BSA conjugates, gray – BSA conjugates, added at day 0. Black (Pos, positive control) – medium alone.

**Figure 3:**
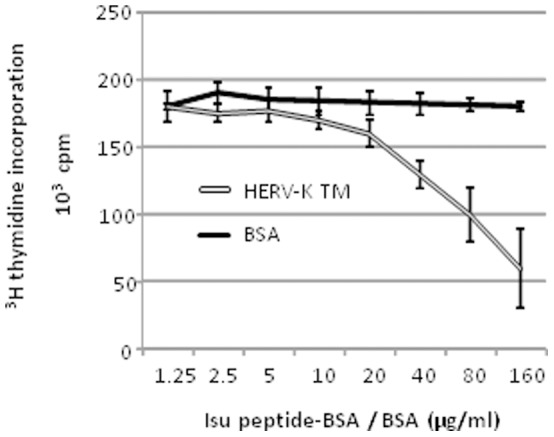
Influence of isu peptide-BSA conjugates on the activation of ConA-stimulated PBMCs from one healthy donor. Cell proliferation was measured by ^3^H-thymidine incorporation. The ^3^H-thymidine was added on day three and cells were then harvested the next day and the counts per minute were determined, black - BSA control, gray – isu peptide-BSA conjugates, added at day 0 (mean±SD; n = 3). It is a representative experiment out of 5.

To study the influence of the TM protein of HERV-K on the activation of human immune cells, the purified glycosylated TM protein produced in yeast cells was added together with the T cell mitogen Concanavalin A (ConA) to PBMCs from healthy human blood donors. A significant inhibition of cell activation was observed when the TM protein of HERV-K was added in comparison to the bovine serum albumin (BSA) and to the medium control as measured by two different methods (Figure 2a, b). This inhibition was dose-dependent. An inhibition was also observed when another T cell mitogen, phytohemagglutinin (PHA), was used (data not shown). Noteworthy, the proliferation assay based on ^3^H-thymidine incorporation was measuring the inhibition more efficiently as compared to the Alamar blue assay. Inhibition of proliferation by the HERV-K TM was also observed in the case of murine splenocytes, indicating an interspecies effect (Figure 2c). Finally, carrier protein-conjugates of the peptide corresponding to the isu domain of the HERV-K TM protein equally lead to a dose-dependent inhibition of cell proliferation (Figure 3), narrowing down the inhibitory effect of the TM protein to this domain.

### The TM Protein of HERV-K Modulated Cytokine Release

**Figure 4:**
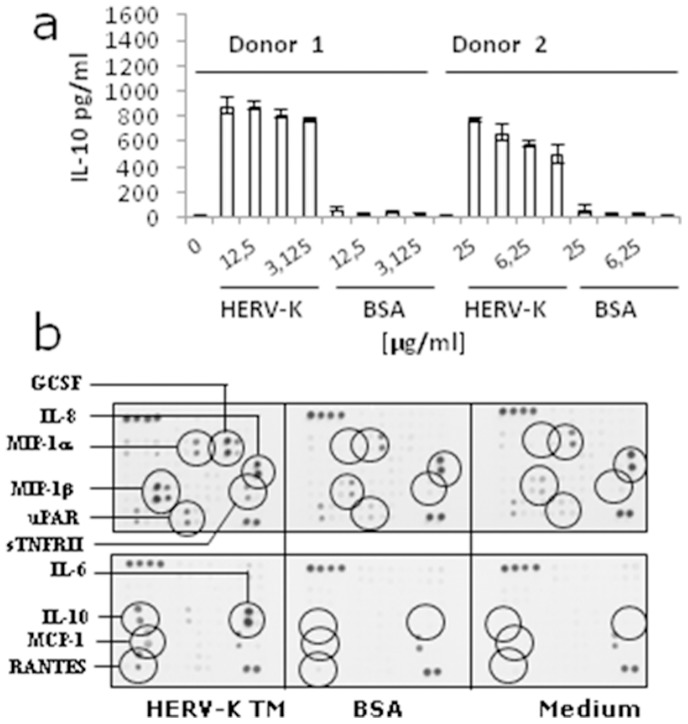
Influence of the HERV-K TM protein on the cytokine release by donor PBMCs. **a,** Dose-dependent induction of Il-10 release in PBMCs from two donors by the TM protein of HERV-K (0– medium control) compared to control protein BSA as measured by ELISAs (mean±SD; n = 3). **b,** Cytokine array measuring simultaneous release of cytokines from human donor PBMCs incubated with the TM protein of HERV-K, or control protein BSA, or medium alone after 24 hrs incubation. The up-regulated cytokines are circled. A list of all analysed cytokines and their full names are given in Table S1.

To analyse the influence of the TM protein of HERV-K on IL-10 release, PBMCs from two healthy human donors were incubated for 24 hrs with different doses of the purified protein and the amount of IL-10 was measured in the supernatant by ELISA (Figure 4a). Release of IL-10 was analysed because in the case of the TM protein gp41 of HIV-1 and the corresponding isu peptide a strong increase in IL-10 release was observed [25], [27]. Further on we analysed the influence of the TM protein of HERV-K on the expression of other cytokines. A microarray assay was performed to study the expression of 62 cytokines (Figure 4b, Table S2). An overexpression was observed for the following cytokines: IL-6, IL-8, IL-10, MCP-1, RANTES, MIP-1alpha, MIP-1beta, uPAR, sTNFRII, GCSF (for the full names and the cytokines with unchanged expression at 24 hrs see Table S2). Similar changes in the cytokine expression were observed when the isu peptide of the TM protein gp41 of HIV-1 was investigated in different assays [28].

### Donor Dependence of the Immunomodulatory Effect by the TM Protein of HERV-K

**Figure 5:**
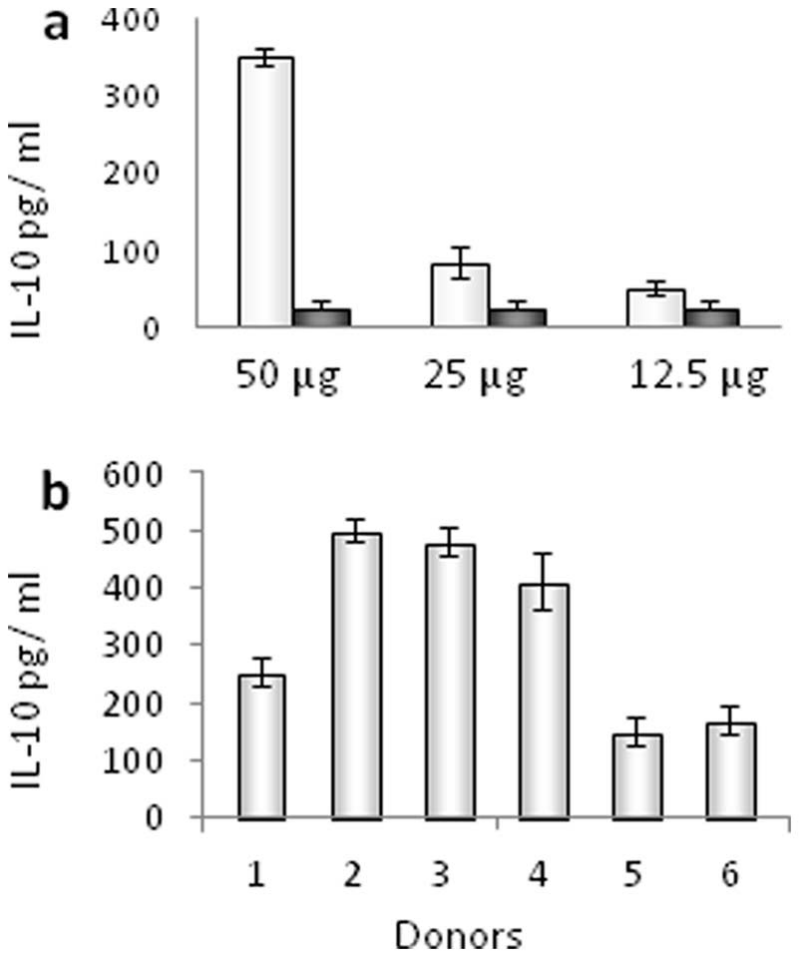
Influence of the homopolymer of the isu peptide of HERV-K on the IL-10 release by donor PBMCs. **a,** Comparison of the isu peptide homopolymer (grey) with a randomised peptide (dark grey). **b,** Comparison of the IL-10 release from PBMCs of six donors treated with one batch and the same amount of the isu peptide homopolymer, the IL-10 release of their PBMCs incubated with medium alone was zero.

When studying the effect of gp41 of HIV-1 and the corresponding isu peptide, some differences in the response among donors were observed [28]. To study this for HERV-K, PBMCs from 6 healthy human donors (5 male, 1 female, 5 Caucasian, 1 African) were incubated with the same batch and the same amount of a polymer preparation of the isu peptide of HERV-K. Reproducible differences in the response were observed (Figure 5). PBMCs from four donors released a high level of IL-10 (above 250 ng/ml), while PBMCs from two donors released less than 150 ng/ml, indicating a variable reactivity of the donors to the isu domain of HERV-K. When PBMCs from a high responder and from a low responder were tested again after 14 days the same differences in IL-10 release were observed.

### The TM Protein of HERV-K Modulated Gene Expression

To study the influence of the TM protein of HERV-K on gene expression in human PBMCs, a genome wide microarray analysis (29.500 genes) was performed. The RNA was isolated from the PBMCs of a healthy blood donor 24 hrs after incubation with the TM protein of HERV-K. In this comparative transcriptome analysis RNA from PBMCs incubated in parallel with medium alone as well as with the isu peptide of the TM protein gp41 of HIV-1 was also analysed. When 2.5 µg TM protein of HERV-K and 12.5 µg isu peptide homopolymer of gp41 of HIV-1 were added to 3×10^5^ cells, both induced release of a similar amount of IL-10 (810 pg/ml).

**Figure 6:**
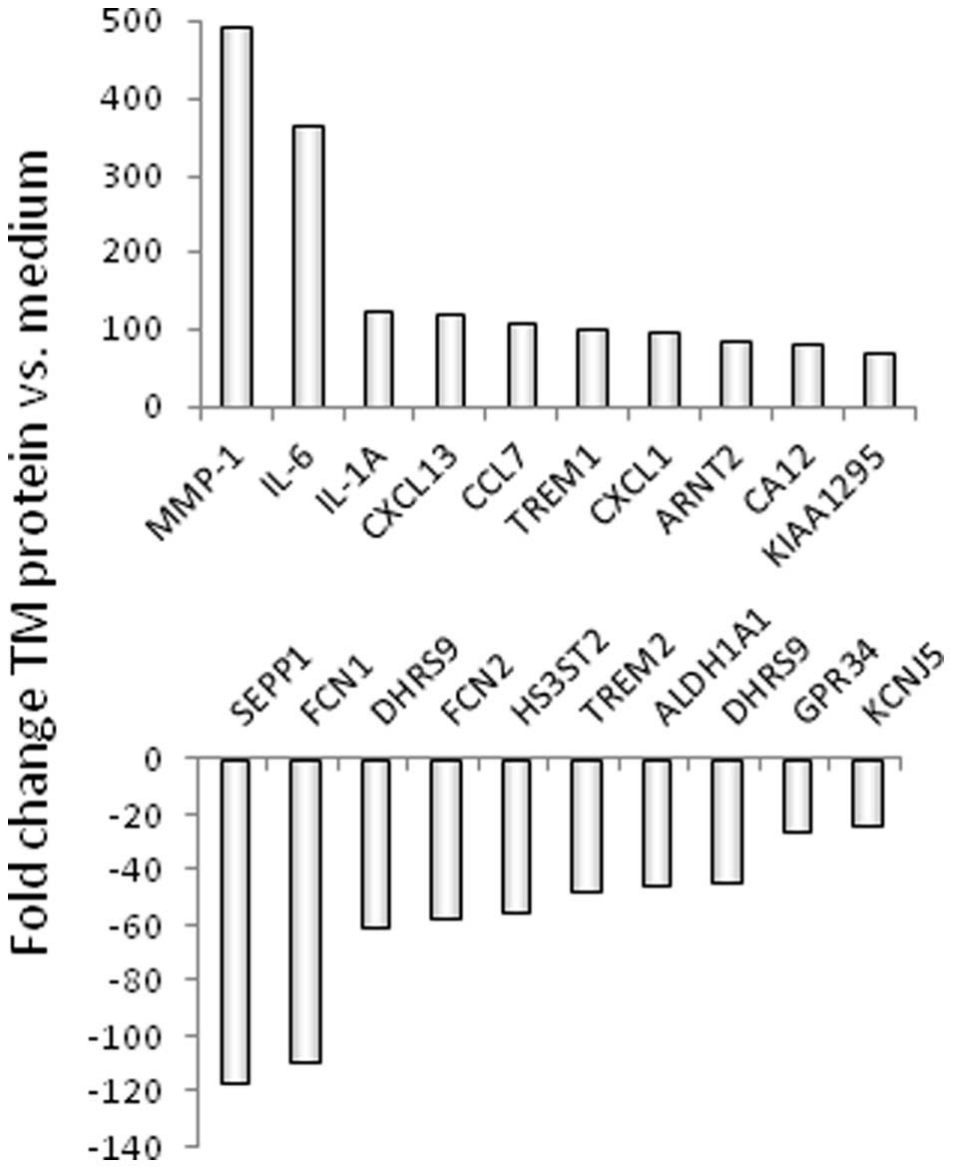
Genes with the highest up-regulation (upper part) or down-regulation (lower part) of their expression in PBMC of one donor in response to the incubation with the TM protein of HERV-K. Fold changes indicate gene expression compared to control cells incubated in medium. The full names of the genes and 40 other up- or down-regulated genes are given in Tables S2 and S3.

More than 300 genes were found up-regulated and more than 300 genes were found down-regulated upon the incubation with the TM protein of HERV-K and the isu peptide of HIV-1. Ten genes of each group (up or down) with the highest fold change after incubation with the TM protein of HERV-K are shown in Figure 6. Fifty genes including the first ten genes with the highest change in expression are shown in Tables S3 (up) and S4 (down).

The expression of the genes up- and down-regulated by the isu peptide of HIV-1 [28] are also shown for comparison in the Tables S3 (up) and S4 (down). Nearly the same genes were up- or down-regulated, however with slight differences. For example, in the case of the isu peptide homopolymer of HIV-1 IL-6 has the first position in the list of the most up-regulated and MMP-1 (matrix metalloproteinase 1) the second, whereas in the case of the TM protein of HERV-K MMP-1 has the first position and IL-6 the second.

As mentioned above, the highest up-regulation induced by the TM protein of HERV-K was shown for the gene of MMP-1 (Figure 6, position 1). MMP-1 is a zinc-dependent protease essential for the breakdown of the extracellular matrix expressed on monocytes and macrophages [29]. The second highest up-regulation was that of the IL-6 gene (Figure 6, position 2), confirming the results on the protein level as shown by the cytokine array (Figure 4b). Interestingly, the genes with higher expression are predominantly involved in two processes “Immunity and defense” and “Signal transduction”. Another up-regulated gene was TREM-1 (triggering receptor expressed on myeloid cells 1) (Figure 6, position 6). TREM-1 has a role as a regulator of innate and adaptive immunity [30], [31].

Among the down-regulated genes were SEPP1 (selenoprotein P plasma 1, position 1 in Figure 6), FCN1 (ficolin, position 2 in Figure 6), as well as FCN2 and TREM2 (position 4 and 6 in Figure 6). These molecules play an important role in innate immune responses [32]–[34].

These results indicate that the recombinant TM protein of HERV-K produced in yeasts modulated the expression of numerous genes in human PBMCs and that this modulation is similar to that induced by the isu domain of the TM protein of HIV-1.

### HERV-K Particles Released from Human Teratocarcinoma Cells Modulated Cytokine Release

**Figure 7:**
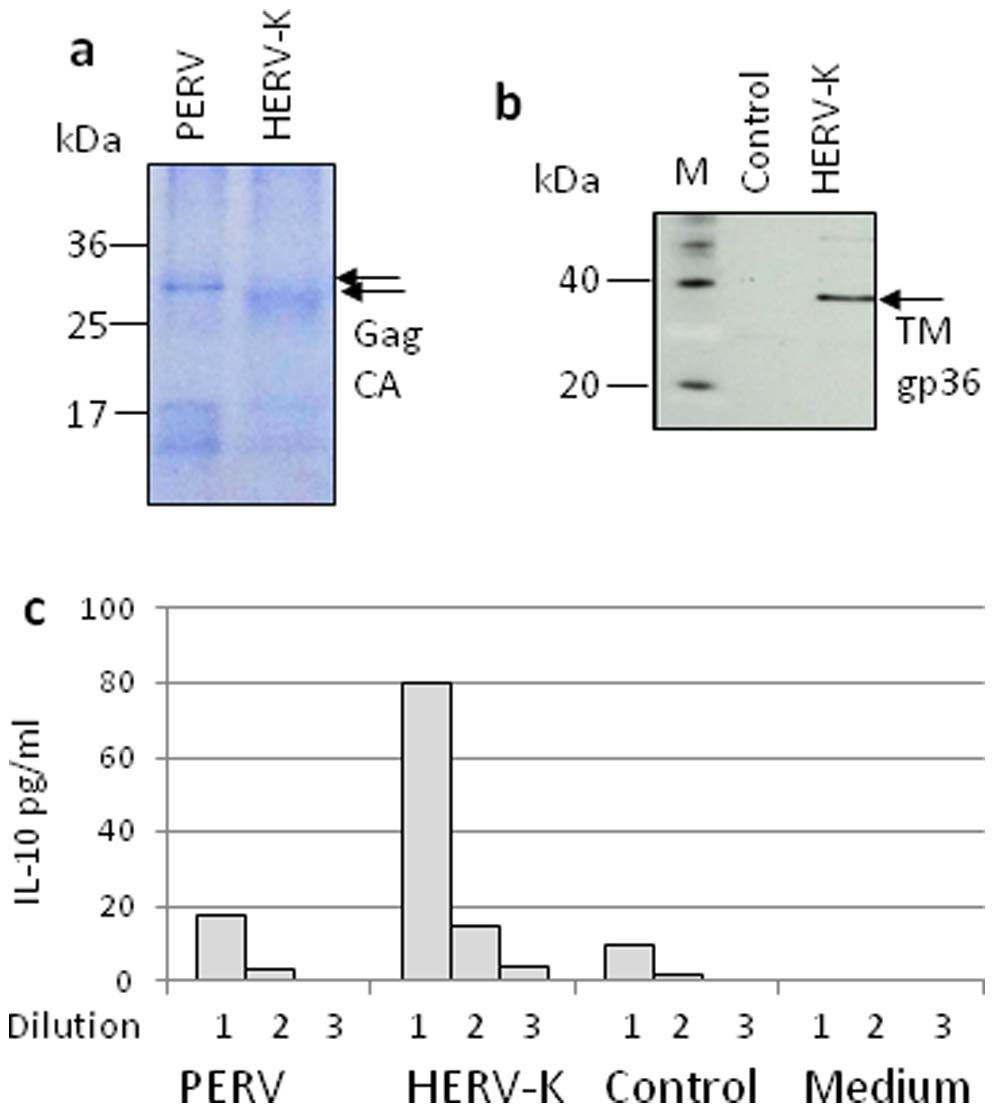
Induction of IL-10 release in human PBMCs by HERV-K and PERV particles. **a,** Characterisation and estimation of the amount of added HERV-K and PERV by SDS-PAGE and Coomassie blue staining. HERV-K containing 60 ng Gag (capsid, CA) and PERV containing 100 ng Gag (capsid, CA) were loaded per lane and the amount of the Gag (CA) proteins was used for comparison. **b,** Detection of the TM protein gp36 of HERV-K in the pellet used for incubation with human PBMCs by a TM specific antiserum, M – marker, control – pelleted supernatant from uninfected 293 cells, HERV-K – pelleted supernatant from GH cells. **c,** IL-10 release by human PBMCs induced by HERV-K particles, by the pellet from the supernatant of uninfected 293 cells corresponding to the same amount of supernatant (control), and by purified PERV particles produced on 293 cells. Virus containing approximately 100 ng Gag (CA) protein, which was used for comparison (see below) and 10 ng TM protein, was added to the first well (1), the dilutions 50/5 ng (2) and 25/2.5 ng (3) are also shown. IL-10 was measured after 24 hrs of incubation.

Human GH germ cells express the TM protein of HERV-K on the cell surface and release pleomorphic non-infectious virus particles [4], [7]. The particles were pelleted and purified by sucrose cushion centrifugation. By Western blot analysis the presence of the TM protein in the virus preparation was shown (Figure 7). Incubation of 3×10^5^ human PBMCs with virus particles containing approximately 10 ng TM protein (for calculation see Materials and Methods) induced the release of 80 pg IL-10/ml (Figure 7). Thus the activity of the virus particles is much stronger than that of the recombinant TM protein produced in yeast cells and that of peptide polymers. In parallel virus particles of the porcine endogenous retrovirus (PERV) were pelleted and assayed. Although approximately the same amount of PERV was added as calculated by similar amounts of their Gag capsid proteins, the induction of IL-10 was lower when compared with that induced by HERV-K (Figure 7).

## Discussion

Most retroviruses exert an immunosuppressive effect when a certain virus load was reached in the infected host. Inactivated retrovirus preparations, their TM proteins and synthetic peptides corresponding to the isu domain of their TM proteins were found to inhibit proliferation of PBMCs and to modulate the cytokine release and gene expression (for review see [25], [26]). Previously we and others had shown that particles of HIV-1 [27], [35], of PERV-A, PERV-B and the murine leukaemia virus (MuLV) [36], of FeLV [37], [38], of the baboon endogenous retrovirus (BaEV), and of a deltaretrovirus [39], [40] as well as of the Koala retrovirus (KoRV) [41] were able to inhibit lymphocyte activation and modulated IL-10 release in treated immune cells.

To extend our studies on the immunosuppressive properties of retroviruses we investigated a human endogenous retrovirus, HERV-K. The influence of virus particles released from a human tumour cell line, of the TM protein and the isu peptide on immune cell proliferation and gene expression was studied using cytokine arrays and transcriptome comparison.

We showed for the first time that HERV-K induced IL-10 release from human PBMCs. We also showed that the recombinant TM protein of HERV-K produced in yeast cells and polymers of a peptide corresponding to the immunosuppressive domain of the TM protein inhibited immune cell activation, and modulated cytokine release and gene expression in human PBMCs.

These data are of importance for the understanding of the role of endogenous retroviruses in carcinogenesis and embryogenesis. Previously we and others had shown that the TM protein of HERV-K is expressed in germ cell tumours and melanomas [11] as well as in the human placenta [12]. The data presented here suggest that the expression of the TM protein may contribute to the suppression of the immune system and thus prevent the rejection of the tumour cells as well as of the embryo, which immunologically represents a semi-allotransplant. In fact, an elevated IL-10 expression was seen in tumour patients as well as in pregnant women [42]–[48]. In particular, plasma IL-10 was steadily increasing during gestation (week 12, p<0.05, week 20, p<0.01 and week 35, p<0.0001, respectively) [42]. Using an antiserum against the TM protein of HERV-K we found expression of this protein in the placenta in villous cytotrophoblast and extravillous cytotrophoblast (EVT) cells invading the decidua [12]. The fact that EVT cells expressing c-erbB2 coding for the human epidermal growth factor (EGF) receptor 2 (ErbB2) [49] are regularly found in intimate contact with maternal immune cells suggests a potential role of HERV-K in the induction of the immunosuppression. When such invasive EVT cells were isolated by ErbB2 affinity chromatography they were found to express the TM protein of HERV-K at a high level [12]. Noteworthily, in the placenta at least three other endogenous retroviruses are expressed, HERV-W, HERV-FRD and ERV-3 [50]–[52]. The envelope protein of HERV-W, which is not immunosuppressive *in vivo,* corresponds to syncytin-1 and the envelope protein of HERV-FRD, which is immunosuppressive *in vivo,* corresponds to syncytin-2 [50]. The envelope protein of HERV-R (ERV3) is also immunosuppressive [51]. Elevated plasma levels of IL-10 were also reported for patients with melanomas [43]–[48]. However it is not quite clear, whether IL-10 is produced by the tumour cells, by the immune cells or both. When we studied the expression of IL-10 in melanoma cell lines with measured expression of the TM protein of HERV-K [11], no IL-10 release was detected in the supernatant (unpublished).

The fact that HERV-K particles, its TM protein and a synthetic peptide corresponding to the isu domain induce the same changes in cytokine expression indicates that the isu domain is the biologically active domain in the virus. Although the isu domain is conserved among retroviruses, there are differences in the sequence of the domain of the gammaretroviruses, of the corresponding sequence of the lentiviruses and that of HERV-K (Figure 1). It is of interest that the sequence of the isu domain of HERV-K is mosaic and contains one amino acids present in all retroviruses, and in addition: one in the gammaretroviruses, one in the lentiviruses and three in MMTV (Figure 1b). It is also important to note that the majority of the HERV-K proviruses, especially those highly expressed in melanomas, e.g., HERV-K6 [53], contains the sequence present in the TM protein and in the peptide used in this study. The importance of this domain was underlined in the case of HIV-1 by mutation in the isu domain. Six mutations abrogated the influence of the isu domain on cytokine release totally, while others not [27]. The amino acids which when mutated abrogated the effect on cytokine release totally are shown in the sequence of the isu domain of HIV-1 in Figure 1 in bold. At least three of them are the most conserved among all retroviruses. The influence of the mutations in the minor sequences of the isu domain of HIV-1 is difficult to predict, however analysis of the conformation using the PROTEAN program showed no significant differences between the variants of HERV-K.

The mechanism of interaction between the isu domain of the TM proteins and the immune cells is still unknown.

First, the conformation of the immunosuppressive domain seems to be important for the immunomodulatory activity. Whereas conjugates of the isu peptide with a carrier protein or homopolymers of the isu peptide are active, the monomer did not show activity [54], [55]. When we compared gp41 of HIV-1 produced in human cells with the homopolymers of the isu peptide of gp41, 700 fold more polymers had to be used to obtain the same release of IL-10 [27]. The difference is certainly due to the conformation of the isu domain in the trimeric gp41 compared with the artificial peptide homopolymer. Differences in the immunomodulatory activity between retroviruses may explain the differences in IL-10 induction between PERV and HERV-K (Figure 7). The slight differences in the expression of cellular genes induced by the isu peptide polymer of HIV-1 on one site and the TM protein of HERV-K on the other also argues in favour of that possibility (Tables S3, S4).

Second, the search for a specific receptor revealed some binding partners [56]–[59]. However, their function in the signal transduction leading to the cytokine modulation is unknown. For gammaretroviruses and HIV several signal transduction pathways were described and an inhibition of the protein kinase C by the isu peptide was shown [60]–[65]. The donor dependence suggests that genetic host factors (receptor polymorphism or differences in signal transduction?) may contribute to the biological activity. It is important to note, that genes involved in innate immunity such as SEPP1, FCN1, FCN2 and TREM2 were downregulated. This allows the virus to manipulate the immune system from the very first moment of infection.

An interesting example of the complex interaction of the isu domain with cells of the immune system is given here in detail. Among the proteins with a higher expression after incubation of human PBMCs with the TM protein of HERV-K are the urokinase-type plasminogen activator receptor (uPAR) and the soluble tumour necrosis factor receptor type II (sTNFRII) (Figure 4b), similarly as it was observed after incubation with the isu peptide of HIV-1 and the TM protein of PERV (unpublished). uPAR is a cell-surface receptor expressed on monocytes and macrophages, which also exists in soluble and cleaved forms. Its ligand, the serine-protease uPA, acts proteolytic on the cell membrane, degrading surrounding extracellular material (ECM). In addition, uPAR can bind with high affinity a component of the ECM, vitronectin, and associate to cell surface molecules such as formyl-peptide receptors (FPR) and integrins to activate signalling pathways inside the cells. This means that uPAR plays an important role in cell proliferation/survival and adhesion/migration, which are crucial events for an efficient immune response to infectious agent [66]. sTNFRII is a soluble variant of the extracellular domain of the receptor II of the tumour necrosis factor (TNF) which is derived from the membrane bound form by the proteolytic activity of a metalloproteinase. sTNFRII is still able to bind to TNF and acts as natural inhibitor of TNF by sequestering soluble TNF and preventing it to bind to its receptor [67]. Interestingly, expression of the metalloproteinase MMP-1 was also increased by the TM proteins of HERV-K (Figure 5) and HIV-1 [28].

Taken together, the previously obtained results and the present study suggest that the immunosuppressive activity may be an intrinsic property of the TM proteins of retroviruses. Further studies searching for a putative receptor as well as investigating the signal transduction pathways leading to the described changes in the cytokine expression should be carried out.

## Materials and Methods

### Cells

The human teratocarcinoma cell line GH, kindly provided by R. Löwer [4], and 293T cells (ATCC CRL11268) were maintained in DMEM supplemented with 10% heat-inactivated fetal calf serum, antibiotics and L-glutamine. 293T cells were infected with stocks of PERV-A/C [68] by spinoculation at 2000 g for 40 min, splitted and supernatant was collected every second day.

### Cloning, Expression in Yeast Cells and Purification of the TM Protein of HERV-K

The ectodomain of the transmembrane envelope protein of HERV-K 108 (which is identical with HML2-HOM, K(C7), ERVK-6, located on chromosome 7p22.1) [10] was re-cloned into the expression vector pFPMT121-MFa-His6-TCS [69] with the signal peptide of the mating factor a (MFa), an inducible promotor element of the methanol oxidase (MOX) gene and a 6 His. Re-cloning was achieved using Not I and Bgl II. The expression vector was transformed into competent yeast cells (*H. polymorpha*) by electroporation. Although the MFa signal peptide was present, the protein was not released into the supernatant. Therefore cells were disrupted by 8 M urea and 0.3% SDS and the protein was purified using His tag affinity chromatography.

### Preparation of Isu Peptide Conjugates

The HERV-K derived isu peptide was produced and conjugated to bovine serum albumin (BSA) as previously described using EDC (1-Ethyl-3-[3-dimethylaminopropyl]carbodiimide hydrochloride) [54]. In addition, the isu peptide (KLANQINDLRQTVIWMGDR) (Figure 1) and a randomised peptide (DILDMRVWKQANGRTLIQN), produced by JPT Berlin, were used for the preparation of homopolymers by cross-linking using EDC and NHS (N-hydroxysuccinimid) (Pierce) as recommended by the supplier.

### Isolation of HERV-K and PERV Particles

Supernatants from GH cells producing HERV-K and 293T cells producing PERV were centrifuged at 1200 g for 10 min and at 4000 g for 15 min. Then the supernatants were filtered through 0.45 mm filters (Millipore), and centrifuged at 28000 rpm (rotor SW32Ti, Beckman, Ireland) for 3 hrs. The pellet was resuspended in PBS and centrifuged through a 20% sucrose cushion at 36000 rpm (rotor SW 50.1Ti, Beckman) for 3 hrs. Virus pellets from initial 100 ml supernatant were resuspended in 50 µl PBS and used immediately or stored at −80°C until use. The amount of TM protein in the preparation was calculated based on the amount of the Gag (capsid, CA) protein using serial dilutions of BSA as standard and the assumption that particles contain approximately 10 times more Gag (CA) than TM protein. Virus preparations and control supernatants from uninfected cells were added to PBMCs after six cycles of freeze-thawing procedure.

### SDS-PAGE, Native PAGE and Western Blot Analysis

Electrophoresis was performed in Tris-Glycine 4%–20% gradient gels using SDS Tris-Glycine sample buffer (Novex, Life technologies Carlsbad, CA, USA). Proteins were transferred onto Protran BA83 0.2 µm membrane (Whatman GmbH, Dassel, Germany) at 45 V for 2 hrs. Membranes were blocked with 6% skimmed milk in PBS with 0.1% Tween 20 (blocking buffer) for 3 hrs at room temperature or overnight at 4°C, incubated for 2 hrs at room temperature with a goat serum against the TM protein of HERV-K [10], [11] (1∶300). After five times (5 min each) washing in PBS with 0.1% Tween 20 (PBS-Tween) the membranes were incubated for 1 h with anti-goat IgG-HRP conjugate (Dako Laboratories, Glostrup, Denmark) diluted 1∶10000 in blocking buffer. The membranes were washed five times (5 min each) in PBS-Tween, treated for 1 min with Pierce ECL Western blotting substrate (Pierce, Rockford, IL, USA) and exposed to CL-XPosure film (Thermo Scientific, USA). The molecular mass was determined using a mixture of the following markers: Page Ruler Plus Prestained Protein Ladder (Fermentas Life Science) and Magic Mark XP Western Protein Standard (Invitrogen).

### Isolation of Human PBMCs and Murine Spleen Cells

Human PBMCs were isolated from whole blood of healthy donors by Ficoll-Hypaque (PAA Laboratories, Austria) density centrifugation using Leucosep tubes (Greiner, Germany). Isolated PBMCs were cultivated with the HERV-K TM protein or BSA at 37°C in RPMI 1640 with 10% fetal calf serum (FCS, Biochrome AG, Berlin, Germany) which had been selected for very low induction of IL-10 in normal PBMCs or medium alone. Spleen cells were obtained from BALB/c mice by mechanical disruption and subsequently washed and incubated in RPMI 1640 with 10% FCS.

### Proliferation Assays

Proliferation assays using ^3^H-thymidine were performed by stimulating 3.25×10^6^ donor PBMCs or murine spleen cells with 10 µg/ml Concanavalin A (ConA, Sigma) in the presence or absence of the HERV-K TM protein or the isu peptide conjugated to BSA. After incubation at 37°C and 5% CO_2_ for 40 hrs ^3^H-thymidine (1 µCi/well) was added and the cells were incubated for additional 24 h at 37°C and then harvested. Counts per minute (cpm) were determined using a Micro Beta Trilux Scintillation and Luminescence counter (Perkin Elmer) or by a 96-well plate beta-counter as described previously [28], [54]. In addition, Alamar blue proliferation assays were performed. 3.25×10^6^ donor PBMCs were incubated with the HERV-K TM protein or with control BSA, with and without 10 µg/ml Concanavalin A (ConA, Sigma). After 48 hrs at 37°C and 5% CO_2_ 20 µl of Alamar blue (Biosource International) were added to each well and after additional incubation for 8 hrs the plates were measured in an ELISA reader Spectra Classic (Tecan) at wavelengths 560 nm and 620 nm. The D values of both results were counted as the specific adsorption.

### Enzyme-linked Immunosorbent Assays for IL-10

Supernatants from donor PMBCs either untreated or treated for 24 hrs with the TM protein of HERV-K were collected by centrifugation at 2000 g for 10 min and tested for IL-10 by ELISA according to the protocol of the supplier (BD Biosciences, San Diego, USA).

### Cytokine Arrays

Cytokine release in the supernatant of treated or untreated donor PBMCs were measured by a membrane-based cytokine array VI (RayBiotech, Inc.) after 24 hrs allowing measurement of 62 different cytokines.

### RNA Isolation from PBMCs

Total RNA was isolated from donor PBMCs using the RNeasy kit (Qiagen, Hilden, Germany). The RNA concentration was measured using a NanoDrop spectrometer ND-100 (PEQLAB), and RNA specimens were used immediately or kept at −80°C before use.

### Microarray

Total RNA was prepared as described above. An RNA integrity numbers (RIN) of 9.1 and 8.8 were determined for the RNA from PBMCs cultured with medium or the TM protein of HERV-K, respectively (RIN 10 is the highest). The microarray was performed by IMGM Laboratories, Munich. 0.5 µg of total RNA were converted into digoxigenin (DIG)-labeled cRNA in a reverse transcriptase in vitro transcription (RT-IVT), 10 µg were fragmented and hybridised using a Human Genome Survey Microarray V2.0 plate from Applied Biosystems. After washing, an anti-DIG-AP-conjugate (Roche, Germany) was applied and signals were detected with an AB1700 Microarray Reader.

### Ethical Statement

The use of human blood has been approved by the ethical commission at the Medical Faculty of the Humboldt University Berlin. Written informed consent was provided by study participants. This study was carried out in strict accordance with the German Animal Protection Act and was approved by the Landesamt für Gesundheit und Soziales (LAGeSo) Berlin. All efforts were made to minimize suffering.

## Supporting Information

Figure S1 Characterisation of the TM protein of HERV-K produced in yeast cells. a, SDS-PAGE of the purified protein stained by Coomassie blue. Arrows indicate the 20, 30 and 32 kDa proteins. b, Western blot analysis using an antibody specific for the His-tag. c, Western blot analysis using a goat serum specific for the TM protein produced in *E. coli.* M ­– marker proteins, 1– TM protein produced in yeast cells and purified by His-tag affinity chromatography. 2– bovine serum albumin, 3 - TM protein produced as calmodulin binding fusion protein in *E. coli*. d, Amino acid sequence of the entire TM protein of HERV-K and the epitopes recognised by the goat serum after immunisation with the TM protein produced in *E. coli*. Blue – sequence of the ectodomain of the TM protein used for immunisation, red – Asn-Xaa-Ser/Thr sequences, green – immunosuppressive domain, underlined – potential glycosylation site according the NetNGlyc 1.0 program, brown – Cys – Cys loop. (TIF)

Table S1 Examples of the sequences of the isu domain of different HERV-K proviruses. Mutations are marked in red. (DOCX)

Table S2 Abbreviations and full names of the cytokines tested for (see Fig. 4). Cytokines with increased expression are shown in red. (DOCX)

Table S3 Results of the microarray analysis: Up-regulated genes. (DOCX)

Table S4 Results of the microarray analysis: Down-regulated genes. (DOCX)
