# Supplementary figures and images for: The Transmembrane Protein of the Human Endogenous Retrovirus - K (HERV-K) Modulates Cytokine Release and Gene Expression
Source: PLoS One. 2013 Aug 7;8(8):e70399. doi: 10.1371/journal.pone.0070399 (PMC3737193; doi:10.1371/journal.pone.0070399)

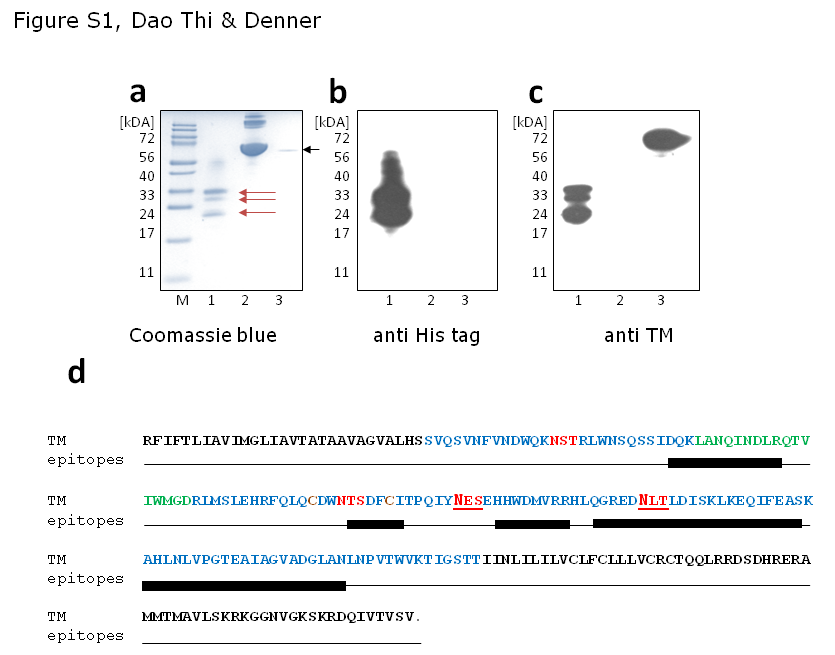

Supplement: Figure S1 — (TIF) [file pone.0070399.s001.tif]
