# Supplementary material for: The Transmembrane Protein of the Human Endogenous Retrovirus - K (HERV-K) Modulates Cytokine Release and Gene Expression
Source: PLoS One. 2013 Aug 7;8(8):e70399. doi: 10.1371/journal.pone.0070399 (PMC3737193; doi:10.1371/journal.pone.0070399)
Supplement: Table S1 — (DOCX) [file pone.0070399.s002.docx]

**Supplementary Table S1**: Examples of the sequences of the isu domain of different HERV-K proviruses

| No | Name | Accession number | Sequence |
| --- | --- | --- | --- |
| 1 | HERV-K chromososme 7 | AAF88168 | lanqindlrqtviw |
| 2 | HERV-K_6q14.1, EnvK4 protein  HERV-K(C6), HERV-K109 | Q9UKH3 | lanqindlrqtviw |
| 3 | HERV-K_1q22, HERV-K(III, HERV-K102, | P61567 | lanqindlrqtviw |
| 4 | HERV-K6, HERV-K_7p22.1, HERV-K(C7), HERV-K(HML-2.HOM), HERV-K108 | Q69384 | lanqindlrqtviw |
| 5 | HERV-K_1q23.3, HERV-K(C1a), HERV-K110, HERV-K18, IDDMK1,2 22 envelope protein | O42043 | lasqindlrqtviw |
| 6 | HERV-K chromosome 7 | AAD21098 | lanqindlrqtviw |
| 7 | HERV-K_5q33.3, HERV-K10, HERV-K107 | P10267 | lanqindlrqtviw |
| 8 | HERV-K expressed in melanoma | AAY87455 | lanqindlrqtviw |
| 9 | HERV-K_16p3.3 | Q9NX77 | lanqindlrqsvtw |
