# Supplementary material for: The Transmembrane Protein of the Human Endogenous Retrovirus - K (HERV-K) Modulates Cytokine Release and Gene Expression
Source: PLoS One. 2013 Aug 7;8(8):e70399. doi: 10.1371/journal.pone.0070399 (PMC3737193; doi:10.1371/journal.pone.0070399)
Supplement: Table S2 — (DOCX) [file pone.0070399.s003.docx]

**Supplementary Table S2**. Abbreviations and full names of the cytokines tested for (see Fig. 4). Cytokines with increased expression are shown in red.

| Abbreviation | Full name |
| --- | --- |
| ANG | Angiogenin |
| BDNF | Brain-derived Neurotrophic Factor |
| BLC | B-lymphocyte Chemoattractant |
| BMP-4 | Bone Morphogenetic Proteins -4 |
| CK β 8-1 | Chemokine beta-8 |
| CNTF | Ciliary Neuronotrophic Factor |
| EGF | Epidermal growth factor |
| Eotaxin | CCL11 |
| Eotaxin-2 | MPIF-2 (Myeloid Progenitor Inhibitory Factor 2 ), CCL24 |
| Eotaxin-3 | MIP-4-alpha (macrophage inflammatory protein-4-alpha ), CCL26 |
| FGF-6 | Fibroblast Growth Factor-6 |
| FGF-7 | Fibroblast Growth Factor-7 |
| Fit-3 Ligand | fms-like Tyrosine Kinase-3 Ligand |
| FKN | Fractalkine |
| GCP-2 | Granulocyte Chemotactic Protein 2 |
| GCSF | Granulocyte Colony-Stimulating Factor |
| GDNF | Glial-derived Neurotrophic Factor |
| GM-CSF | Granulocyte macrophage colony-stimulating factor |
| GRO | Growth Related Oncogene |
| GRO-α | Growth Related Oncogene-Alpha |
| I-309 |  |
| IFN-γ | Interferon-gamma |
| IGFBP-1 | Insulin-like Growth Factor Binding Proteins 1 |
| IGFBP-2 | Insulin-like Growth Factor Binding Proteins 2 |
| IGFBP-4 | Insulin-like Growth Factor Binding Proteins 4 |
| IGF-I | Insulin-like growth factor-1 |
| IL-10 | Interleukin-10 |
| IL-13 | Interleukin-13 |
| IL-15 | Interleukin-15 |
| IL-16 | Interleukin-16 |
| IL-1α | Interleukin-1 alpha |
| IL-1β | Interleukin-1 beta |
| IL-1rα | Interleukin-1 receptor alpha |
| IL-2 | Interleukin-2 |
| IL-3 | Interleukin-3 |
| IL-4 | Interleukin-4 |
| IL-5 | Interleukin-5 |
| IL-6 | Interleukin-6 |
| IL-7 | Interleukin-7 |
| IL-8 | Interleukin-8 |
| lep. | Leptin |
| LIGHT | an acronym derived from: homologous to **l**ymphotoxins, **i**nducible expression, competes  with HSV **g**lycoprotein D for **H**VEM, a receptor expressed on **T**-lymphocytes |
| MCP-1 | Monocyte Chemoattractant Protein 1 |
| MCP-2 | Monocyte Chemoattractant Protein 2 |
| MCP-3 | Monocyte Chemoattractant Protein 3 |
| MCP-4 | Monocyte Chemoattractant Protein 4 |
| M-CSF | macrophage colony stimulating factor; monocyte colony stimulating factor |
| MDC | Macrophage-derived Chemokine |
| MIG (CXCL9) | Monokine induced by Gamma Interferon |
| MIP-1α | Macrophage Inflammatory Protein 1 Alpha |
| MIP-1β | Macrophage Inflammatory Protein 1 Beta |
| MIP-1δ | Macrophage Inflammatory Protein 1 Delta |
| MIP-3α | Macrophage Inflammatory Protein 3 alpha |
| NAP-2 | Neutrophil Activating Peptide 2 |
| NT-3 | Neurotrophin-3 |
| PARC | Pulmonary and Activation-Regulated Chemokine |
| PDGF-BB | Platelet-derived Growth Factor BB |
| RANTES | Regulated upon activation T-cell expressed and presumably secreted |
| SCF | Stem Cell Factor |
| SDF-1 | Stromal Cell-derived Factor |
| TARC | Thymus and Activation-Regulated Chemokine |
| TGF-β 1 | Tumor Necrosis Factor beta-1 |
| TGF-β 3 | Tumor Necrosis Factor beta-3 |
| TNF-α | Tumor necrosis factor-alpha |
| TNF-β | Tumor necrosis factor-beta |
| uPAR | Urokinase-type plasminogen activator receptor |
| sTNFRII | Soluble Tumor Necrosis Factor Receptor Type II |
