# Supplementary material for: The Transmembrane Protein of the Human Endogenous Retrovirus - K (HERV-K) Modulates Cytokine Release and Gene Expression
Source: PLoS One. 2013 Aug 7;8(8):e70399. doi: 10.1371/journal.pone.0070399 (PMC3737193; doi:10.1371/journal.pone.0070399)
Supplement: Table S3 — (DOCX) [file pone.0070399.s004.docx]

| **Supplementary Table S3.** Results of the microarray analysis: Up-regulated genes | | | | | |
| --- | --- | --- | --- | --- | --- |
|  | | | | | |
| **Position** | **Abbreviation** | **Full name** | **FC** | **FC HIV** | **Position HIV** |
| 1 | MMP1 | matrix metallopeptidase 1 | 493.02 | 597.54 | 2 |
|  |  | (interstitial collagnase) |  |  |  |
| 2 | IL6 | interleukin 6 (interferon, beta 2) | 363.36 | 809.07 | 1 |
| 3 | IL1A | interleukin 1, alpha | 121.83 | 196.47 | 3 |
| 4 | CXCL13 | chemokine ligand 13 | 118.71 | 108.02 | 10 |
| 5 | CCL7 | chemokine (C-C motif) ligand 7 | 107.30 |  |  |
| 6 | TREM1 | triggering receptor expressed on | 100.48 | 94.92 | 11 |
|  |  | myeloid cells 1 |  |  |  |
| 7 | CXCL1 | chemokine ligand 1 | 97.79 | 122.59 | 9 |
| 8 | ARNT2 | aryl-hydrocarbon receptor | 84.89 | 51.32 | 18 |
|  |  | nuclear translocator 2 |  |  |  |
| 9 | CA12 | carbonic anhydrase XII | 80.86 | 54.63 | 16 |
| 10 | KIAA1295 | KIAA1295 | 68.81 | 52.34 | 17 |
| 11 | PTGS2 | prostaglandin-endoperoxide synthase 2 | 59.47 | 146.41 | 7 |
| 12 | IL1B | interleukin 1, beta | 56.88 | 64.25 | 13 |
| 13 | IL24 | interleukin 24 | 55.75 | 130.58 | 8 |
| 14 | INHBA | inhibin, beta A | 35.33 | 81.86 | 12 |
| 15 | CSPG2 | chondroitin sulfate proteoglycan 2 | 34.99 | 18.69 | 45 |
| 16 | AQP9 | aquaporin 9 | 34.68 | 24.90 | 35 |
| 17 | IL8 | interleukin 8 | 34.53 | 41.98 | 22 |
| 18 | TNIP3 | TNFAIP3 interacting protein 3 | 33.79 | 23.38 | 38 |
| 19 | TM4SF1 | transmembrane 4 L six family member 1 | 31.58 | 9.93 | 77 |
| 20 | CXCL3 | chemokine (C-X-C motif) ligand 3 | 31.49 | 43.93 | 20 |
| 21 | PI3 | peptidase inhibitor 3, skin-derived | 30.27 | 36.84 | 24 |
| 22 | MCEMP1 |  | 29.79 | 22.99 | 40 |
| 23 | NDP | Norrie disease (pseudoglioma) | 25.99 | 22.95 | 41 |
| 24 | DNER |  | 24.32 | 23.18 | 39 |
| 25 | C1QTNF1 | C1q and tumor necrosis factor | 23.16 | 25.78 | 33 |
|  |  | related protein 1 |  |  |  |
| 26 | EREG | epiregulin | 22.88 | 42.16 | 21 |
| 27 | RETN | resistin | 22.44 | 15.77 | 52 |
| 28 | GOS2 | G0/G1switch 2 | 22.15 |  |  |
| 29 | INSM1 | insulinoma-associated 1 | 21.12 | 31.55 | 28 |
| 30 | MMP12 | matrix metallopeptidase 12 | 20.95 | 36.71 | 25 |
| 31 | EMR3 | egf-like module containing, | 20.84 | 20.28 | 43 |
|  |  | mucin-like, hormone receptor-like 3 |  |  |  |
| 32 | FPRL1 | formyl peptide receptor-like 1 | 19.91 | 13.20 | 59 |
| 33 |  |  | 18.92 |  |  |
| 34 | LOC541472 |  | 18.45 | 11.82 | 63 |
| 35 | PTGES | prostaglandin E synthase | 17.69 | 36.51 | 26 |
| 36 | PBEF1 | pre-B-cell colony enhancing factor 1 | 17.31 | 14.21 | 57 |
| 37 | SLC16A10 | solute carrier family 16 | 17.24 | 16.63 | 51 |
| 38 | PDPN | podoplanin | 16.39 | 10.06 | 76 |
| 39 | CCL20 | chemokine (C-C motif) ligand 20 | 16.32 | 46.36 | 19 |
| 40 |  |  | 16.16 |  |  |
| 41 | CXCL6 | chemokine (C-X-C motif) ligand 6 | 15.98 | 40.50 | 23 |
| 42 | THBD | thrombomodulin | 15.55 | 25.48 | 34 |
| 43 | IL1F9 | interleukin 1 family, member 9 | 13.79 | 56.59 | 15 |
| 44 | CYP27B1 | cytochrome P450, family 27, | 13.77 | 7.51 | 92 |
|  |  | subfamily B, polypeptide 1 |  |  |  |
| 45 | PROCR | protein C receptor, endothelial | 12.99 | 10.90 | 69 |
| 46 | SERPINB7 | serpin peptidase inhibitor, | 12.77 | 27.38 | 30 |
|  |  | clade B, member 7 |  |  |  |
| 47 | MMP14 | matrix metallopeptidase 14 | 12.59 | 17.39 | 48 |
| 48 | MAOA | monoamine oxidase A | 12.54 | 10.09 | 75 |
| 49 | TFPI | tissue factor pathway inhibitor | 12.36 | 10.85 | 71 |
| 50 | SLC11A1 | solute carrier family 11 (proton-coupled |  |  |  |
|  |  | divalent metal ion transporters), member 1 | 12.18 | 8.49 | 81 |

The position of the gene according to the fold changes (FC) value, the FC values for the expression in cells incubated with the TM protein of HERV-K, the FC vakues for the expression in cells incubated with the isu peptide of HIV-1, and the corresponding position in the experiment with the HIV-1 isu peptide are shown.
