# Supplementary material for: The Transmembrane Protein of the Human Endogenous Retrovirus - K (HERV-K) Modulates Cytokine Release and Gene Expression
Source: PLoS One. 2013 Aug 7;8(8):e70399. doi: 10.1371/journal.pone.0070399 (PMC3737193; doi:10.1371/journal.pone.0070399)
Supplement: Table S4 — (DOCX) [file pone.0070399.s005.docx]

| **Supplementary Table S4.** Results of the microarray analysis: Down-regulated genes | | | | | |
| --- | --- | --- | --- | --- | --- |
|  | | | | | |
| **Position** | **Abbreviation** | **Full name** | **FC** | **FC** | **Position** |
|  |  |  |  | **HIV** | **HIV** |
| 1 | SEPP1 | selenoprotein P, plasma, 1 | -116.31 | -93.46 | 3 |
| 2 | FCN1 | ficolin (collagen/ |  |  |  |
|  |  | fibrinogen domain containing) 1 | -109.01 | -188.73 | 1 |
| 3 | DHRS9 | dehydrogenase/ |  |  |  |
|  |  | reductase (SDR family) member 9 | -60.51 | -94.62 | 2 |
| 4 | FCN2 | ficolin (collagen/fibrinogen |  |  |  |
|  |  | domain containing lectin) 2 (hucolin) | -57.28 |  |  |
| 5 | HS3ST2 | heparan sulfate (glucosamine) |  |  |  |
|  |  | 3-O-sulfotransferase 2 | -55.10 | -48.17 | 8 |
| 6 | TREM2 | triggering receptor expressed |  |  |  |
|  |  | on myeloid cells 2 | -46.95 | -71.56 | 4 |
| 7 | ALDH1A1 | aldehyde dehydrogenase 1 family, |  |  |  |
|  |  | member A1 | -45.51 |  |  |
| 8 | DHRS9 | dehydrogenase/reductase |  |  |  |
|  |  | (SDR family) member 9 | -43.88 | -37.09 | 12 |
| 9 | GPR34 | G protein-coupled receptor 34 | -25.51 | -30.66 | 15 |
| 10 | KCNJ5 | potassium inwardly-rectifying channel, |  |  |  |
|  |  | subfamily J, member 5 | -23.95 |  |  |
| 11 | MS4A6A | membrane-spanning 4-domains, |  |  |  |
|  |  | subfamily A, member 6A | -21.71 | -41.81 | 10 |
| 12 | CXCL10 | chemokine (C-X-C motif) ligand 10 | -20.23 | -56.34 | 5 |
| 13 | MS4A6E | membrane-spanning 4-domains, |  |  |  |
|  |  | subfamily A, member 6E | -19.32 | -41.81 | 10 |
| 14 | FGL2 | fibrinogen-like 2 | -17.42 | -45.37 | 9 |
| 15 | FABP3 | fatty acid binding protein 3, muscle and |  |  |  |
|  |  | heart (mammary-derived growth inhibitor) | -16.65 | -16.23 | 25 |
| 16 | CLEC10A | C-type lectin domain family 10, member A | -16.47 |  |  |
| 17 | PMFBP1 | polyamine modulated |  |  |  |
|  |  | factor 1 binding protein 1 | -15.03 |  |  |
| 18 | CD36 | CD36 antigen (collagen type I receptor, |  |  |  |
|  |  | thrombospondin receptor) | -14.84 | -54.52 | 7 |
| 19 | CAMP | cathelicidin antimicrobial peptide | -14.60 | -25.76 | 17 |
| 20 | ADORA3 | adenosine A3 receptor | -14.59 | -13.26 | 28 |
| 21 |  |  | -14.04 |  |  |
| 22 | TSPAN4 | tetraspanin 4 | -13.39 | -19.02 | 21 |
| 23 | CXCL9 | chemokine (C-X-C motif) ligand 9 | -12.18 | -17.67 | 22 |
| 24 | RNASE1 | ribonuclease, RNase A family, 1 (pancreatic) | -11.43 | -34.05 | 13 |
| 25 | GPNMB | glycoprotein (transmembrane) nmb | -10.95 | -40.14 | 11 |
| 26 | RARRES1 | retinoic acid receptor responder |  |  |  |
|  |  | (tazarotene induced) 1 | -10.63 | -8.03 | 48 |
| 27 | FUCA1 | fucosidase, alpha-L- 1, tissue | -10.54 | -33.82 | 14 |
| 28 | TLR7 | toll-like receptor 7 | -10.39 |  |  |
| 29 | DEFA4 | defensin, alpha 4, corticostatin | -10.25 |  |  |
| 30 | TCN2 | transcobalamin II; macrocytic anemia | -9.72 |  |  |
| 31 | ASRGL1 | asparaginase like 1 | -9.61 | -13.65 | 27 |
| 32 | FLJ22662 |  | -9.32 |  |  |
| 33 | CD302 | CD302 antigen | -8.58 | -11.98 | 30 |
| 34 | EPHB2 | EPH receptor B2 | -8.52 | -16.46 | 23 |
| 35 | STARD13 | START domain containing 13 | -8.36 | -7.84 | 50 |
| 36 | TLR5 | toll-like receptor 5 | -8.36 | -8.02 | 49 |
| 37 | IGSF2 | immunoglobulin superfamily, member 2 | -8.29 | -9.35 | 41 |
| 38 | HLA-DMB | major histocompatibility complex, |  |  |  |
|  |  | class II, DM beta | -7.86 | -8.47 | 46 |
| 39 | F13A1 | coagulation factor XIII, A1 polypeptide | -7.59 | -8.63 | 45 |
| 40 | CD9 | CD9 antigen (p24) | -7.28 | -6.97 | 63 |
| 41 | FOLR2 | folate receptor 2 (fetal) | -7.25 |  |  |
| 42 | FXYD6 | FXYD domain containing ion |  |  |  |
|  |  | transport regulator 6 | -7.25 | -7.05 | 62 |
| 43 |  |  | -7,.09 |  |  |
| 44 | LIPA | lipase A, lysosomal acid, cholesterol |  |  |  |
|  |  | esterase (Wolman disease) | -7.09 | -7.28 | 56 |
| 45 | ADORA3 | adenosine A3 receptor | -6.99 | -13.26 | 28 |
| 46 | CD163L1 | CD163 antigen-like 1 | -6.79 | -10.37 | 35 |
| 47 | CST6 | cystatin E/M | -6.77 |  |  |
| 48 | LGMN | legumain | -6.49 | -10.36 | 36 |
| 49 | LRRN3 | leucine rich repeat neuronal 3 | -5.99 | -5.40 | 91 |
| 50 | MNDA | myeloid cell nuclear differentiation antigen | -5.97 |  |  |

The position of the gene according to the fold changes (FC) value, the FC values for the expression in cells incubated with the TM protein of HERV-K, the FC vakues for the expression in cells incubated with the isu peptide of HIV-1, and the corresponding position in the experiment with the HIV-1 isu peptide are shown.
